# The risk of cardiovascular events in the Italian adult population without prior history of CVD: a systematic review of follow-up studies

**DOI:** 10.3389/fcvm.2026.1645953

**Published:** 2026-04-29

**Authors:** Ilaria Valentini, Maddalena Arcelli, Camilla Gobbetti, Francesca Volpi, Lorenzo Conciarelli, Stefania Boccia, Chiara de Waure

**Affiliations:** 1 Department of Medicine and Surgery, University of Perugia, Perugia, Italy; 2 Section of Hygiene, University Department of Life Sciences and Public Health, Università Cattolica del Sacro Cuore, Rome, Italy; 3 Department of Woman and Child Health and Public Health, Fondazione Policlinico Universitario A. Gemelli IRCCS, Rome, Italy

**Keywords:** cardiovascular disease, coronary artery disease, incidence, Italy, lethality, long-term follow-up, stroke, systematic review

## Abstract

**Background:**

Cardiovascular diseases (CVDs) remain the leading cause of mortality and morbidity in Europe. Understanding their burden is essential to planning effective public health responses. However, in Italy, comprehensive data on the incidence and lethality of major cardiovascular events, including myocardial infarction and stroke, are fragmented and heterogeneous. This review aims to assess the incidence of fatal and non-fatal major cardiovascular events in Italian adults without prior CVD.

**Methods:**

A systematic review was conducted according to the PRISMA 2020 guidelines and registered on PROSPERO (CRD42025624126). We searched PubMed and Web of Science to identify longitudinal studies involving Italian adults aged 18–65 years without baseline CVD. Data extracted from the selected studies included study design, population characteristics, follow-up, and results. Quality was assessed using the Newcastle-Ottawa Scale. The results were synthesised narratively and stratified by cardiovascular event and study design.

**Results:**

Nineteen studies were included, with a follow-up of 1–50 years. The incidence of coronary heart disease ranged from 10‰ per year to cumulative values as high as 46.5‰ over 40 years. The incidence of stroke ranged from 1.6‰ to 2.75‰ per year, with early lethality (28–30 days after the event) between 18.1% and 33%. Variability in estimates was associated with differences in study design, baseline population characteristics, endpoints definition, calendar period and geographical context.

**Conclusions:**

This systematic review highlights the enduring burden of CVD in Italy in the short- and long-term horizon. Although incidence and lethality have declined in recent decades, the findings underscore the importance of ongoing surveillance and tailored prevention strategies to control CVD in Italy.

**Systematic Review Registration:**

https://www.crd.york.ac.uk/PROSPERO/view/CRD42025624126, PROSPERO CRD42025624126.

## 1 Introduction

Cardiovascular diseases (CVD) remain the leading cause of mortality, morbidity, and disability in Europe, accounting for 45% of all deaths (1–3). This poses a substantial burden on public health systems and economies also because CVD account for a substantial share of premature deaths, namely those occurring before an age cut-off that, albeit not unequivocally defined, ranges from 50 to 75 years (4). CVD encompass a range of heart and blood vessel disorders, including coronary heart disease (CHD), which in the literature is also referred to as ischemic heart disease (IHD) or coronary artery disease (CAD), as well as cerebrovascular disease, peripheral artery disease, and others (5). In this manuscript, we consistently use CHD as an umbrella term, as it includes myocardial infarction (MI), angina, coronary artery stenosis, and heart failure. As for cerebrovascular diseases, they often manifests as ischemic stroke or transient ischemic attack (TIA) (6, 7).

Like Europe, Italy shows high CVD-related mortality and morbidity (8). CVD account for around 35% of total deaths in Italy, with an age-standardized mortality rate of 113 per 100,000 population (3, 8). CHD and stroke, the two principal manifestations of CVD, rank as the first and second most common causes of death both globally and in Italy (3, 9). Specifically, CHD account for 15.5% of total deaths and contributes 6.8% to the morbidity burden, while stroke accounts for 9.5% of deaths and 4.3% of morbidity (8). CVD represent a significant burden also economically and in terms of public health, accounting for approximately 7.6% to 21.0% of national healthcare expenditure, mainly due to CHD and stroke, with the largest share due to hospital inpatient care and a smaller one (3%–7%) due to pharmacological treatments (10). In addition, CVD are responsible for a substantial share of disability-adjusted life years (DALYs) lost in Italy, equal to 16.0% of the total (age-standardized rate: 1,763.8 per 100,000 population). This is slightly higher than the global estimate of 14.7% (age-standardized rate: 1,459.8 per 100,000 population) (8). Therefore, the burden of CVD is still high, notwithstanding the decline in mortality and DALYs observed since 1990 (11).

Despite the critical burden of CVD, up to 90% of cases can be prevented by taking necessary preventive interventions (12). According to a World Health Organization (WHO) report, reducing cardiovascular mortality effectively requires focusing on three key areas: surveillance (monitoring and mapping the epidemic of CVD), prevention (reducing exposure to risk factors), and management (ensuring equitable healthcare for people with CVD) (12). Prevention strategies are categorized into three levels: *primary*, *secondary* and *tertiary prevention* (12, 13). In Italy, as in many other countries, efforts to reduce the burden of CVD have mostly relied on secondary prevention strategies aimed at individuals with existing conditions. However, most cardiovascular events occur in individuals with no prior history of CVD and are responsible of premature deaths. In this light, primary prevention strategies are of utmost importance, especially in younger people. The current literature highlights various effective approaches to lower CVD risk, with a strong consensus on the need to raise awareness about CVD risk factors, the often asymptomatic progression of the disease, and the significant influence of healthy behaviours and lifestyle choices (14–16). This underscores the vital importance of prioritizing primary prevention and early identification of risk factors within the general population as early as possible.

Follow-up studies offer valuable information on the incidence and lethality of cardiovascular events, allowing the monitoring of CVD epidemiology, which is a complementary action to prevention. Although several large-scale international studies have contributed to assess the incidence of CVD and identify key risk factors, data specific to the Italian context (5, 15–18), particularly among adult individuals without a history of CVD, remain fragmented and heterogeneous.

Few systematic reviews or European reports provided comprehensive estimates of CVD-related mortality and morbidity in Europe (3, 19–21) but they missed to report data stratified by countries and by individual characteristics, such as prior CVD.

This review addresses this gap by synthesising longitudinal follow-up studies conducted on the Italian population to evaluate the risk of cardiovascular events in the adult population without previous CVD. In line with a CVD prevention-oriented rationale (22), we focused on adults up to 65 years of age to capture the avoidable burden that can be effectively reduced through primary prevention and early risk identification, before cardiovascular risk becomes largely driven by advanced age, multimorbidity, and less reversible biological processes. Specifically, our review aims to examine the incidence of fatal and non-fatal major cardiovascular events in the adult population without CVD to support and guide public health interventions.

## 2 Methods

### 2.1 Study design

This systematic review was conducted to evaluate the risk of developing a major cardiovascular event in the Italian adult population without prior history of CVD. The research protocol was registered with PROSPERO, the International Prospective Register of Systematic Reviews (PROSPERO: CRD42025624126) (23). Our methods and the reporting of results were strictly in line with the recommendations provided in the Preferred Reporting Items for Systematic Reviews and Meta-Analyses (PRISMA) 2020 guidelines (24). We also adhered to the methodological standards set forth in the Cochrane Handbook for Systematic Reviews of Interventions (25).

### 2.2 Search strategy

A comprehensive literature search, using an electronic search strategy, was performed on PubMed and Web of Science. The last search was conducted on March 5th, 2024.

The search strategy was based on controlled vocabulary (MeSH terms), synonyms, related terms, and free terms. The complete search strategies are provided in the Supplementary Material. We applied language restrictions, including only studies published in English or Italian. No limitations were imposed on the publication date.

### 2.3 Eligibility criteria

All identified articles were reviewed according to criteria defined according to PEO (Population, Exposure, Outcome + Study Design) (26) (Table 1). Eligible studies included adults over 18 years of age. We excluded studies that exclusively enrolled older populations (i.e., >65 years). This age restriction reflects a prevention-oriented rationale (22), as the adult population represents the primary target for primary prevention strategies aimed at preventing the onset of CVD. Therefore, the exclusion of older age groups was a deliberate methodological choice rather than an attempt to minimise the epidemiological relevance of CVD at older ages.

**Table 1:** PEOS study selection criteria.

Domain	Inclusion	Exclusion
Population	Studies focusing on the Italian adult population without a prior history of CVD.	Enrolled participants with a history of CVD. Population under 18 years or over 65 years of age. Enrolled only people with known cardiovascular risk conditions (e.g., diabetes mellitus). Involved non-Italian populations.
Exposure	Exposed to the risk of developing a CVD	
Outcome(s)	Any new cardiovascular event (primary endpoint). Any consequence, such as such as hospitalization and death, related to the cardiovascular event (secondary endpoint)	Studies not reporting any outcome relevant to the review objective
Study design	Longitudinal studies either prospective or retrospective, published in English or Italian	Reviews or meta-analyses

### 2.4 Study selection

Duplicate entries were first removed. After this step, the selection process was carried out in two stages. In the first stage, the titles and abstracts of the studies were evaluated based on the eligibility criteria. In the second stage, the full texts of the studies deemed eligible during the initial screening were thoroughly reviewed to confirm their inclusion in the systematic review.

The selection process was conducted independently by two researchers at each stage of the study selection. Any disagreements were resolved through discussion with a third reviewer making the final decision if necessary.

### 2.5 Data extraction

Data from the studies included in the systematic review were extracted and organised using an excel table created by the researchers. This table was used to collect, structure and summarise key information from the selected studies.

The following details were extracted: title, authors and publication date, study design, setting (regions), sample size, gender and age distribution, recruitment period, median follow-up period, inclusion criteria, study objective, study population characteristics (including the distinction between cohort studies and population-based studies using record linkage), assessed endpoints with their definition, and results for each endpoint. Data on the incidence of all cardiovascular events (primary endpoint) and related consequences (secondary endpoints) were extracted as risk (percentage) or rate (per person-time). Data stratification was conducted by fatal and non-fatal events, as well as by type of cardiovascular event. In case of studies conducted in the general population, only data related to the adult population were extracted. Similarly, only data related to the Italian population were extracted from studies conducted in more countries.

### 2.6 Quality assessment

The quality of the included studies was assessed based on the Newcastle-Ottawa Scale (NOS) (27, 28), which consists of three sections. The studies were classified according to the total score as follows: 5 or less: poor quality; 5–6: medium quality, 7–9: high quality. All studies were included regardless of quality, but the latter was considered in the qualitative synthesis of the results.

### 2.7 Strategy for data synthesis

Data synthesis combined narrative and tabular approaches to provide a comprehensive and structured summary of the findings of included studies. Key study characteristics, such as publication year, study population characteristics, study design, setting, follow-up duration, type of endpoints assessed, and data collection methods, were presented narratively and supported by a single table. In addition, a second summary table was used to systematically present the incidence of cardiovascular events, distinguishing between fatal, non-fatal, and combined (total) events. The results were further categorised according to the type of cardiovascular event to facilitate cross-study comparison.

## 3 Results

### 3.1 Literature search results

**Figure 1:**
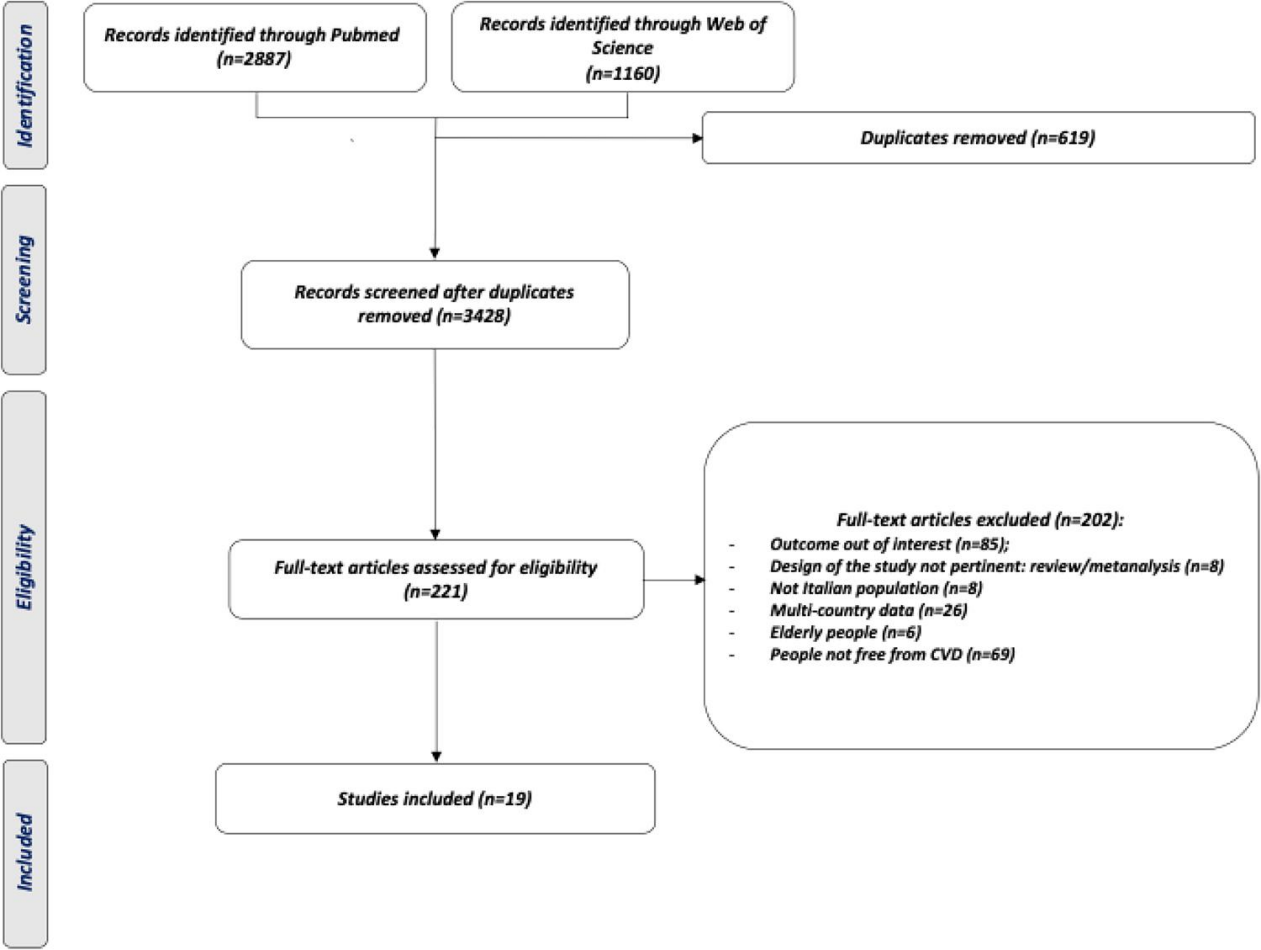
PRISMA diagram for inclusion and exclusion of the study.

Figure 1 presents the flow diagram of the study selection process. Initially, searches in electronic databases yielded 4,047 studies. After removing duplicates, 3,428 studies remained for further examination. The selection of titles and abstracts led to the exclusion of 3,207 studies, resulting in 221 full-text articles retrieved for detailed evaluation. Finally, a total of 19 studies were included.

### 3.2 Study characteristics

The included studies (*n* = 19) (29–47) were published between 1984 and 2023 and most of the publications were from the last two decades. Supplementary Table S1 reports a summary of the characteristics of the study.

Three studies (29, 46, 47) included in the review were derived from the EPICOR cohort, which focused on cardiovascular epidemiology and dietary patterns in various European regions. In particular, two studies (46, 47) shared the same objective but differed in their follow-up duration. Additionally, a study (43) originated from the EPIC study, a larger cohort that is part of the EPICOR project, and analysed the relevance of dietary and lifestyle factors in cardiovascular health.

Seven studies (35, 38–42, 45) were based on cohorts from the renowned Seven Countries Study, a landmark investigation of the relationship between diet, lifestyle, and CVD in different populations and duration of follow-up (35, 38, 39).

The majority of studies (*n* = 15, 78.9%) were multi-center (29, 30, 33, 35–43, 45–47) whereas four (21.1%) were single-center (31, 32, 34, 44).

Eight studies have stood out for their population-based approach, which means that they included as study sample the entire resident population of a defined geographical area. These studies were conducted by linking records from multiple sources of health information, including disease registries, demographic databases, and hospital discharge records. Two of these studies focused on CHD (34, 44) while six addressed stroke (30–33, 36, 37).

The studies covered 12 out of 20 Italian regions (60%), with a balanced geographical distribution: Emilia-Romagna and Marche, *n* = 7 (36.8%); Piemonte, *n* = 6 (31.6%); Lombardia, Toscana, Lazio, and Campania, *n* = 4 (21.1%); Valle D'Aosta, *n* = 2 (10.5%); Veneto, Abruzzo, and Puglia, *n* = 1 (5.3%). Additionally, four studies (21.1%) included all three geographical macro-areas (North, Center, and South Italy).

The sample sizes varied widely, ranging from 1,677 to 1,539,784 participants, depending on the type of study (multicenter or not, population based or not). The age ranges of the included population also differed between studies, with seven studies (36.8%) focusing on individuals aged 40–59 years (35, 38–42, 45), five studies (26.3%) examining participants aged 35–74 years (29, 43, 44, 46, 47), one study (5.3%) including individuals aged 25–50 years (34), one study (5.3%) focusing on participants over 37 years (33), and five studies (26.3%) encompassing all age groups (30–32, 36, 37).

Regarding sex distribution, seven studies (36.8%) included only males (35, 38–42, 45), one study (5.3%) included only females (34), and 11 studies (57.9%) included both sexes (29–33, 36, 37, 43, 44, 46, 47), with the proportion of males ranging from 30% to 49.5% and females from 50.5% to 70%.

The duration of follow-up varied significantly, from 1 to 50 years, and half of the studies had a follow-up longer than 10 years, providing a comprehensive long-term perspective on cardiovascular risk.

In respect to endpoints, six cohort studies (35, 38–40, 45, 46) and two population-based studies (34, 44) evaluated the incidence and mortality of CHD; eight studies the incidence of stroke (29–33, 36, 37, 47), including two cohort studies (29, 47) and six population-based studies (30–33, 36, 37); the remaining three studies, all cohort studies, assessed the incidence of various major cardiovascular events (MCV) (41–43). The reported results included mortality from cardiovascular events in five studies (26.3%) (35, 38, 39, 42, 43); the total incidence of cardiovascular events (both fatal and non-fatal) in two studies (10.5%) (44, 47), and the incidence of non-fatal cardiovascular events in 11 studies (57.9%) (29–34, 36, 37, 40, 41, 47). Only one study (5.2%) reported the incidence of non-fatal cases and total incidence as well as mortality (45).

Data collection was based on record linkage in all 19 studies (100%), with three studies (34, 37, 44) (15.8%) relying exclusively on this method. Anamnestic data were collected through questionnaires or interviews with patients, family members, and/or caregivers in 11 studies (57.89%) (29, 35, 38–43, 45–47). In eight studies (42.10%) (30, 31, 35–39, 42) data were collected through interviews with healthcare professionals and periodic patient reassessments during the follow-up.

### 3.3 Quality assessment

Table 2 provides an overview of the NOS scores of the included studies. Five studies were rated as high quality (9/9), three as high-to-moderate quality (8/9), seven as moderate quality, of which six and one respectively scored 7/9 and 6/9. The four remaining studies received lower scores (≤5/9).

**Table 2:** Newcastle–Ottawa quality assessment scale (NOS) for cohort studies.

Author	Selection				Comparability of cohorts based on the design or analysis	Outcome			Final score
	Representativeness of the exposed cohort	Selection of the non-exposed cohort	Ascertainment of exposure	Demonstration that outcome of interest was not present at start of study		Assessment of outcome	Was follow-up long enough for outcomes to occur	Adequacy of follow-up of cohorts	
Sieri et al., (46)^a^	★	★	★	★	★★	★	★	★	9/9
Agnoli et al., (29)^a^	★	★	★	★	★★	★	★	★	9/9
Sieri et al., (47)^a^	★	★	★	★	★★	★	★	★	9/9
Pala et al., (43)^a^	★	★	★	★	★★	★	★		8/9
Keys et al., (35)^b^	★	★	★	★	★★	★	★	★	9/9
Menotti et al., (38)^b^	★	★	★	★	★★	★	★	★	9/9
Menotti et al., (42)^b^	★	★	★	★	★★	★	★		8/9
Menotti et al., (39)^b^	★	★	★	★	★★	★	★		8/9
Menotti et al, (40)^b^		★	★	★	★	★	★	★	7/9
Puddu and Menotti, (45)^b^		★	★	★	★	★	★	★	7/9
Menotti and Menotti, (41)^b^		★	★	★	★	★	★	★	7/9
D’Alessandro et al., (32)	★	★		★	★				4/9
Lauria et al., (36)	★	★		★	★	★			5/9
Carolei et al., (30)	★	★		★	★	★			5/9
Petrelli et al, (44)	★	★	★	★	★	★		★	7/9
Manobianca et al., (37)		★	★	★	★	★	★		6/9
Corso et al., (31)	★	★		★	★	★			5/9
D'Ovidio et al., (34)	★	★	★	★	★	★	★		7/9
De Bont et al., (33)		★	★	★	★★		★	★	7/9

a EPICOR study.

b Seven Country study.

The studies with the maximum ratings (29, 35, 38, 46, 47) demonstrated excellent cohort representativeness, robust exposure ascertainment, and proper follow-up. These studies, particularly those from the Seven Countries Study and the EPICOR study, provided detailed adjustments for confounders and near-complete follow-up, ensuring the reliability of their findings. Notably, other three studies showed high quality receiving a score of 8/9 (39, 42, 43) showing robust design features but some flaws across cohorts and follow up.

The six moderate quality studies scored as 7/9 (33, 34, 40, 41, 44, 45) showed robust methodological designs but presented some limitations related to exposure assessment, outcome measurement across cohorts, and follow-up completeness. The study with a score of 6/9 (31) showed additional limitations, particularly concerning the representativeness of the exposed cohort.

In contrast, four studies were scored low (30, 32, 36, 37) primarily due to limited follow-up periods, incomplete outcome assessments, or lack of robust adjustments for confounders. These limitations highlight potential biases and restrict the generalisability of their findings. All these studies were population-based studies reporting the yearly incidence of stroke.

### 3.4 Outcome measurement

### 3.4.1 Coronary heart disease

CHD was the most common endpoint considered with six cohort studies (35, 38–40, 45, 46), and two population-based studies that addressed the incidence and lethality of CHD (34, 44). Table 3 summarizes extracted data on CHD incidence and lethality.

**Table 3:** Incidence of CHD.

Type Study	Author	Fatal Events	Non-Fatal Events	Total Events
COHORT STUDIES	Sieri et al, (46)^a^	–	–	^a^10 per 1,000 person-years
	Keys et al. (35)^b^	^c^Crevalcore: 42.5 per 1,000 after 15 years of follow-up Montegiorgio: 44.7 per 1,000 after 15 years of follow-up Rome: 51.6 per 1,000 after 15 years of follow-up -	–	–
	Menotti et al. (38)^b^	^c^Crevalcore: 129 per 1,000 after 25 years of follow-up Montegiorgio: 113 per 1,000 after 25 years of follow-up Italy (total): 122 per 1,000 after 25 years of follow-up	–	–
	Menotti et al. (39)^b^	^c^Italy (total): 128 per 1,000 after 25 years of follow-up	–	–
	Menotti et al. (40)^b^	–	–	Incidence over 50 years of follow-up (by age 90): CHD 28.8% HDUE 17.7% ^c^Combined CHD + HDUE 46.5%
	Puddu et al. ^a^(45)^b^	–	Incidence over 50 years of follow-up: CHD: 1.5% HDUE: 2.9%	Incidence over 50 years (%): CHD 26.9% HDUE 20.6% ^c^Combined CHD + HDUE 47.5%
POPULATION-BASED STUDIES	Petrelli et al. (44)	^a^Men: 0.62 per 1,000 person-years ^a^Women:0.084 per 1,000 person-years	–	^c^Men: 5.28 per 1,000 person-years Women: 0.96 per 1,000 person-years
	D'Ovidio et al. (34)	Women ^a^0.146 per 1,000 women over 8 years. ^a^0.0183 per 1,000 person-years	–	0.402 per 1,000 person-years

a EPICOR study.

b Seven Country study.

c Data elaborate by the Authors.

CHD, Coronary Heart Disease; HDUE, Heart Disease of Uncertain Etiology.

In particular, five of the eight studies (62.5%) were part of the Seven Countries Study project (35, 38–40, 45). Because these analyses relied on the same underlying cohort and comparable methodology, differences in reported estimates are likely attributable primarily to variations in follow-up duration rather than to differences in study design or population characteristics. Within this cohort, cumulative mortality from CHD increased with longer follow-up, with age-adjusted mortality rates ranging from 42.5‰ in Crevalcore, 44.7‰ in Montegiorgio, and 51.6‰ in Rome after 15 years of follow-up (35) to approximately 122–128‰ after 25 years of follow-up (38, 39).

Long-term follow-up analyses further showed that cumulative CHD incidence reached approximately 26.9–28.8% after 50 years (40, 45). These estimates were consistent across studies because of the shared population base and methodological framework of the Seven Countries Study. When heart diseases of uncertain etiology (HDUE) were included, cumulative incidence increased to approximately 46.5–47.5% after 50 years (40, 45), highlighting the broader long-term burden of cardiac disease. Puddu et al. (45) also reported the cumulative incidence of non-fatal CHD (1.5% after 50 years of follow-up) (45).

Additional evidence was provided by the EPICOR cohort, which reported a crude annual CHD incidence of approximately 10 per 1,000 person-years (46). This figure is not directly comparable to the long-term cumulative incidence estimates reported in the other studies, as EPICOR provides an annual event rate from a contemporary cohort, whereas the Seven Countries Study and the population-based investigations report cumulative risks accrued over much longer follow-up periods. These differences in time scale and underlying population characteristics likely explain why the EPICOR estimate appears lower than the others.

Population-based studies offered complementary insights. Both investigations were conducted in the Marche region, but included substantially different populations, which likely explains the observed differences in incidence estimates. Petrelli et al. (44) examined a mixed and relatively older population and reported higher incidence rates, particularly among men (5.28 per 1,000 person-years) compared with women (0.96 per 1,000 person-years), as well as higher fatal CHD incidence in men (0.62 per 1,000 person-years) than in women (0.084 per 1,000 person-years) (44). In contrast D'Ovidio et al. (34) focused exclusively on a younger female population and reported substantially lower incidence estimates, including 0.402 per 1,000 person-years for total CHD events and 0.146‰ for fatal events over eight years of follow-up (34). These differences likely reflect the combined influence of age structure and sex composition of the study populations.

#### 3.4.2 Stroke

A total of eight studies investigated the incidence of stroke (29–33, 36, 37, 47) comprising two cohort studies (29, 47) and six (30–33, 36, 37) population-based studies, representing 31.6% of the total (see Table 4). Cohort studies, particularly those from the EPICOR project, reported the number of cases for each type of stroke without analysing regional or gender differences. Population-based studies, on the other hand, provided broader insights.

**Table 4:** Incidence of stroke.

Type Study	**Author**	**Fatal Events**	**Non-Fatal Events**	Total Events	Lethality
COHORT STUDIES	Agnoli et al.^a^ (29)		**–**	^b^3.95‰ over 7.89 years	**-**
	Sieri et al.^a^ (47)	^b^0.66‰ over 10.9 years	^b^7.34‰ over 10.9 years	^b^8‰ over 10.9 years	**-**
POPULATION-BASED STUDIES	D’Alessandro et al. (32)	**–**	**–**	First Stroke Episode – Crude Incidence: Total: 2.23‰ per year By Gender: 1.98‰ per year (males), 2.46‰ per year (females) Age-Adjusted Incidence: Total: 2.15‰ per year By Gender: 2.48‰ per year (males), 1.99‰ per year (females)	31% at 30 days
	Lauria et al. (36)	**–**	**–**	First Stroke Episode—Crude Incidence: Total: 2.24 ‰ per year By Gender: 2.01‰ per year (males), 1.45‰ per year (females)	33% at 30 days
	Carolei et al. (30)	**–**	**–**	First Stroke Episode—Crude Incidence: Total: 2.75 ‰ per year By Gender: 2.76‰ per year (males), 2.74 ‰ per year (females)	
	Manobianca et al. (37)	**-**	**–**	First Stroke Episode—Crude Incidence: Total: 1.60 ‰ per year By Gender: 2.00‰ per year (males), 1.30 ‰ per year (females)	18.1% at 28 days
	Corso et al. (31)	**–**	**–**	First Stroke Episode—Crude Incidence: Total: 2.23 ‰ per year By Gender: 2.24‰ per year (males), 2.23 ‰ per year (females)	19% at 28 days 32% at 1 year
	J. De Bont et al. (33)	**-**	**–**	**^b^**First Stroke Episode—Crude Incidence: Total: 2.67‰ per year	–

^a^ Seven Country study.

^b^ Data elaborate by the Authors.

Cohort studies reported a crude incidence of total stroke ranging from 3.95‰ over 7.89 years (29), to 8‰ over 10.9 years (47). Sieri et al. (47) reported an incidence of non-fatal events and fatal events of 7.34‰ and 0.66‰ respectively over 10.9 years.

In population-based studies (30–33, 36, 37, 42), the crude annual incidence of the first stroke episode ranged from 1.6‰ (37) to 2.75‰ per year (30), with most studies clustering around 2.2‰ to 2.5‰ per year (31, 32, 36). For example, D'Alessandro et al. (32) reported an age-adjusted incidence of 2.15‰ per year, with higher rates in males (2.48‰ per year) compared to females (1.99‰ per year). Similarly, Lauria et al. (36) found a total crude incidence of 2.24‰ per year, with 2.01‰ per year for males and 1.45‰ per year for females, reflecting the higher burden of stroke in male populations. A comparable pattern was observed by Manobianca et al. (37) who reported a lower overall incidence of 1.60‰ per year but again with higher rates among males (2.00‰ per year) compared with females (1.30‰ per year). By contrast, Corso et al. (31) found almost identical risks in males and females (2.24‰ vs. 2.23‰ per year), a pattern similar to that reported by Carolei et al. (30) who also documented nearly identical risks between men (2.76‰ per year) and women (2.74‰ per year). De Bont et al. (33) contributed with the most recent estimates, reporting a crude annual incidence of first-ever stroke of 2.67‰. This finding further supports the consistency of stroke incidence estimates in large population-based studies in Italy across a wide time window ranging from 1992 to 2023.

Among the included studies, lethality following a first stroke episode was reported primarily in population-based studies, with considerable variability depending on the period of observation (i.e., 28–30 days or 1 year). D' Alessandro et al. (32) reported a 30-day lethality rate of 31%, while Lauria et al. (36) observed a slightly higher rate of 33% within the same time window. Carolei et al. (30) provided both short- and long-term data, indicating a lethality of 25.6% at 30 days and 36.9% at 1 year, suggesting significant post-stroke mortality beyond the acute phase. Corso et al. (31) also observed a lethality of 32% at 1 year, consistent with the long-term estimate from Carolei et al. (30).

Manobianca et al. (37) and Corso et al. (31) reported lower short-term lethality rates, with 18.1% and 19% at 28 days, respectively. These lower estimates could be due to the improvement of stroke management as the two studies were published long after the others.

#### 3.4.3 Major cardiovascular events

Table 5 presents a synthesis of the data extracted for MCV from three cohort studies: Pala et al. (43), Menotti et al. (42), and Menotti et al. (41). The cumulative incidence of MCV varied significantly in these studies, highlighting differences in population characteristics and duration of follow-up.

**Table 5:** Incidence of MCV.

Type Study	**Author**	**Fatal Events**	**Non-Fatal Events**	**Total Events**
COHORT STUDIES	Pala et al.^a^ (43)	10.2‰ after 15 years of follow-up for MCV^b^	–	–
	Menotti et al. (42)^b^	Major CHD: 64.7‰ after 10 years of follow-up Stroke: 31.6‰ after 10 years of follow-up ACVD: 100‰ after 10 years of follow-up	–	–
	Menotti et al. (41)^b^	Major CHD: 127‰ after 25 years of follow-up Stroke: 28‰ after 25 years of follow-up for.	Over a 50-year follow-up, 53.9% of men experienced a MCV before the age of 90.	Cumulative incidence peaked at 223‰ after 25 years of follow-up for major CHD and 105‰ after 25 years of follow-up for stroke

a EPICOR study.

b The definition of MCV varies across studies.

CHD, Coronary Heart Disease; ACVD, All Cardiovascular Diseases; MCV, Major Cardiovascular Events.

Menotti et al. (42) reported a cumulative incidence of fatal events at 10 years of follow-up of 64.7‰ for major CHD, 31.6‰ for stroke, and 100‰ for total atherosclerotic cardiovascular disease (ACVD). In the same cohort, Menotti et al. (41) reported results at both 25 and 50 years of follow-up. After 25 years, the cumulative incidence was 223‰ for major CHD and 105‰ for stroke. Over 50 years of follow-up, 53.9% of the cohort experienced at least one major cardiovascular event (fatal or non-fatal).

In contrast, Pala et al. (43) observed a cumulative incidence of fatal MCV of 10.2‰ after 15 years of follow-up, which is substantially lower than Menotti studies estimates (41, 42). However, the cohort enrolled in the three studies were different in respect of birth date with Pala et al. (43) considering a most recent cohort. This, together with the fact that Menotti (41, 42) enrolled only men, could explain the lower incidence of fatal MCV. Eventually, Menotti et al. (41) highlighted the important role of both fatal and non-fatal events in contributing to the overall disease burden (Table 5).

## 4 Discussion

Despite many advances in cardiovascular care and in contrasting CVD mortality, CHD and stroke remain major causes of illness and death both globally and in Italy (48). This systematic review provides a comprehensive synthesis of follow up studies that assessed the incidence of cardiovascular events in the Italian adult population without previous CVD.

CHD was one of the most frequently investigated outcomes, with incidence estimates varying widely depending on study design and follow-up duration. In long-term cohort studies, cumulative incidence reached approximately 26%–29% over 50 years of follow-up and increased to about 46%–47% when cardiac diseases of unknown aetiology were included, while population-based studies generally reported lower annual incidence rates and a higher burden among men compared with women. Stroke incidence estimates were consistent across studies, particularly in population-based investigations, which reported crude annual incidence ranging from approximately 1.6‰ to 2.75‰, with most estimates clustering around 2.2‰–2.5‰ per year. Several studies reported higher incidence among men, although sex differences were not always consistent. Short-term lethality following a first stroke event varied considerably across studies, ranging from approximately 18% to 33% within 28–30 days, while one-year lethality approached one-third of cases. Evidence on MCV also indicated a considerable long-term disease burden. Longitudinal cohort studies reported cumulative incidence values exceeding 200‰ for major CHD and 100‰ for stroke after 25 years of follow-up, with more than half of participants experiencing at least one major cardiovascular event during extended follow-up.

The most elevated incidence estimates were reported in older cohort studies, notably those originating from the Seven Countries Study (35, 38–42, 45), with follow-up periods up to 50 years, homogenous male-only populations, and participants enrolled as early as the 1960s. These cohorts captured a population likely exposed to more cardiovascular risk factors, with limited access to primary prevention and less effective therapeutic options, thus accumulating a higher number of fatal and non-fatal events over time. Conversely, more recent studies, such as those from the EPICOR study (29, 43, 46, 47) and population-based studies (30–34, 36, 37, 44), conducted after the 1990s, with shorter follow-up durations, mixed-sex or female-only samples, and often a healthier baseline population, reported substantially lower incidence values.

The overall pattern emerging from Italian studies is broadly consistent with contemporary European and international data. In recent large-scale primary prevention cohorts, CHD incidence was generally reported in the low single-digit range per 1,000 person-years. For example, in a large Spanish population-based cohort study from January 2007 until December 2014, overall CHD incidence was 3.47‰ (49), with higher rates in men than in women. Similarly, in the multinational Global Cardiovascular Risk Consortium, which included 128,973 participants recruited between July 2000 and May 2019 across 26 countries of different income levels age- and sex-standardised CVD incidence rate was 4.1 per 1,000 person-years; furthermore, 41% of events occurred in cardiovascular disease-naive participants classified as at low risk (50), confirming that a substantial proportion of events occurs in populations free of previous CVD. Compared to other high-income countries, Italy records lower CHD mortality than Northern Europe and the United States, a trend often described as the “Mediterranean paradox” (51), that is a relatively lower mortality despite the presence of CHD risk factors and a high intake of saturated fat. The paradox may be related to the role of the Mediterranean diet, which has been shown to reduce overall mortality (−8%) and CVDs incidence and/or mortality (−10%) (52).

A similar interpretation applies to stroke. The Italian estimates collected in this review are compatible with evidence from Western and Southern Europe. In the German LuSSt registry, conducted between January 2006 and December 2007, the incidence of first-ever stroke was 1.46‰ (52). Data from the population-based Dijon Stroke Registry (France), collected between January 2013 and December 2020, reported a stroke incidence of 236.9 per 100,000 person-years, providing further evidence of comparable stroke incidence (53). For stroke, Europe shows striking geographic disparities, with age-adjusted mortality and DALY rates up to twelve times higher in Eastern than in Western countries (54). Italy, similar to other Mediterranean settings, reports comparatively low incidence and mortality, yet the absolute number of events is increasing with population ageing.

Consistent with national and international literature (55–59), our results confirm that gender is as key dimensions of heterogeneity in the burden of CVD. Across the studies, males generally tend to exhibit higher age-standardised incidence for most CVD, while an opposite trend is observed for crude incidence rates. This phenomenon can be explained by the advancing age of women, whose cardiovascular risk increases after menopause, although it does not fully reach the risk level observed in men (58). In addition, women's higher life expectancy results in their over-representation in older age groups, where the risk of CVD is naturally higher. Consequently, this leads to higher crude incidence rates in older women (59). These findings reinforce the importance of implementing gender-specific prevention strategies, as well as promoting more nuanced approaches to risk assessment and clinical decision-making.

An important consideration in interpreting our findings is the methodological heterogeneity of included studies. Although most of the investigations were multicenter and encompassed several Italian regions, ensuring comparable procedures for data collection and measurement of the outcome, a subset of monocentric or single-region studies relied almost exclusively on record linkage. These population-based designs, conducted within circumscribed geographical areas, captured nearly the entire resident population and integrated information from multiple administrative and clinical registries, minimising the risk of selection bias. By contrast, multicenter and international cohorts, such as those of the Seven Countries Study and EPICOR, offered larger sample sizes, extended follow-up periods, and more comprehensive adjustment for potential confounders, although they sometimes lacked granularity of information. In terms of methodological quality, multicenter studies generally achieved higher NOS scores, reflecting stronger representativeness and exposure assessment. By contrast, monocentric population-based studies, typically conducted in single regions and based primarily on record linkage, while relatively strong in terms of complete follow-up, were more frequently limited by shorter observation periods, less extensive confounder adjustment, and consequently lower NOS scores. In general, these differences indicate that although the methodological rigour was overall medium-high, the consistency of cardiovascular risk estimates in Italian regions can be affected by the type of study design and its relative robustness. Nevertheless, it should also be observed that the studies reporting lower NOS scores investigated the burden of stroke in single Italian regions and in a short-time frame providing consistent epidemiological estimates. Furthermore, their results were also aligned with those from a multicentre European study with longer follow up and high quality.

Looking ahead, to fully understand and respond to the evolving burden of CVD in Italy, future efforts should prioritise the creation of integrated real-time surveillance systems, the linkage of electronic health records, and the longitudinal monitoring of population risk. Only through a coordinated and harmonised national approach high-quality and actionable data can be provided to support evidence-based public health decisions and optimise the allocation of resources in cardiovascular care and prevention. It is well-known that the decline in CVD mortality observed in Italy over the past decades reflects the combined effects of risk factor control (e.g., hypertension, cholesterol, smoking) and therapeutic innovations (60, 61), but does not eliminate the urgency to act on persistent and emerging lifestyle-related risk factors. In fact, as also observed in broader international analyses (11), the combination of population ageing and improved survival determine that the absolute number of individuals living with CVD still remains high. The 2019 ACC/AHA Guideline on the Primary Prevention of Cardiovascular Disease emphasizes that fostering a healthy lifestyle from an early age is the most effective strategy to prevent CVD (22). This approach includes engaging in regular physical activity (62), maintaining a healthy weight, adopting a balanced diet (63), and smoking cessation (14, 64). Several countries have introduced national prevention strategies. In the UK, for example, the NHS Health Check prevention programme (65) launched in 2009, targets adults aged 40–74 without pre-existing cardiovascular disease, diabetes, or kidney disease. It is delivered primarily in primary care settings and aims to identify people at increased risk of cardiovascular disease, type 2 diabetes, kidney disease, and stroke. The programme includes an assessment of risk factors (such as blood pressure, cholesterol, body mass index, smoking, and family history) and provides tailored lifestyle advice or referral to preventive interventions when indicated (65). Such policies do not exist in isolation; they complement a broader mix of legislative and structural actions across Europe. Norway provides a long-standing example: its sugar tax, introduced decades ago, has since been followed by similar policies in Finland, Estonia, France, Ireland, the UK, Spain, and Poland (66) to reduce the consumption of sugar-sweetened beverages and promote healthier dietary choices. The evidence of these initiatives suggests measurable benefits in reducing cardiovascular risk, highlighting the potential value of adapting similar systematic approaches in Italy to complement existing strategies. Effectively addressing modifiable risk factors often demands the implementation of behavioural strategies, which highlights the crucial role of interdisciplinary collaboration among healthcare professionals (67–69).

The results of this review must be interpreted considering certain limitations. The included studies show methodological variability, which limits their comparability and prevent statistical combination of extracted data. In addition, the literature search was restricted to PubMed and Web of Science, which, although the main databases, do not cover all potentially relevant sources and this might contribute to the risk of publication bias. The exclusion of grey literature and studies published in languages other than English or Italian may have introduced publication bias, potentially omitting relevant data, although our focus was on studies conducted in the Italian population, that less likely can be published in other languages. Eventually, older cohort studies reflect healthcare setting and risk profiles that differ markedly from the current situation. It is therefore important to consider the risk of overestimation arising from such studies; similarly, it should be bear in mind that the findings could move away from available statistics because the review focused only on adults instead of the overall population.

Despite its limitations, this review presents several important strengths. It provides a first long-term epidemiological overview of CVD epidemiology in the general asymptomatic adult Italian population, a group often under-represented in favour of clinical or high-risk cohorts. In addition, the inclusion of cohort and population-based studies, conducted in different historical periods, allows a nuanced interpretation of temporal trends. This is of interest considering the absence of national-level continuous surveillance system that hinder the ability to monitor trends in a timely and integrated manner. Eventually, the methodological quality of the included studies was overall medium-high.

## 5 Conclusion

This systematic review gathers data from longitudinal studies conducted in Italy in a 30-year time span. The findings show that the incidence of CHD and stroke in Italian adults without prior CVD, albeit declined over time, remains epidemiologically relevant, particularly when interpreted in light of population ageing. The heterogeneity across studies estimates reflects both true temporal changes and methodological differences.

These results highlight the critical role of primary prevention strategies in mitigating this public health challenge. More standardised and continuously updated national surveillance data are needed to better monitor cardiovascular risk in the Italian population and to support prevention strategies grounded in CVD epidemiology, which reflects the complex interplay of demographic, lifestyle, and healthcare factors. Future efforts should focus on generating more comparable data on specific population subgroup to enable advancement of personalised prevention.

## Data Availability

The original contributions presented in the study are included in the article/Supplementary Material, further inquiries can be directed to the corresponding author.
