# Supplementary material for: The risk of cardiovascular events in the Italian adult population without prior history of CVD: a systematic review of follow-up studies
Source: Front Cardiovasc Med. 2026 Apr 29;13:1645953. doi: 10.3389/fcvm.2026.1645953 (PMC13167500; doi:10.3389/fcvm.2026.1645953)
Supplement: Supplementary file 1 [file Table1.docx]

# Supplemental Material 1

Table S1 - Characteristics of Included Studies

| **Author and Year** | **Type of Study** | **Country/Region** | **Sample Size** | **Recruitment period** | **Follow up duration in years** | **Inclusion criteria** | **Study population mean age At recruitment** | **% males** | **Study’s**  **objective** |
| --- | --- | --- | --- | --- | --- | --- | --- | --- | --- |
| Sieri et al., 2010 (42)* | Prospective Multicenter Cohort  Study | European Study “EPIC”: Denmark, France, Germany, Greece, Netherlands, Spain, Norway, Sweden, United Kingdom, Italy: Varese (Lombardia), Torino (Piemonte), Firenze (Toscana), Napoli (Campania), Ragusa (Sicilia) | Initial: 47,749, with 15,171 males and 32,578 females (Varese: 12,083; Turin: 10,604; Florence: 13,597; Ragusa: 6,403; Naples: 5,062)  Investigated: 44,132, with 13,637 males and 30,495 females | 1993-98 | 7,9 (mean) | 1.Free from MCV, 2. 35-74 years old | 54,5 | 30,90% | To investigate the relationship between glycemic index and glycemic load with the risk of coronary artery disease |
| Sieri et al., 2013 (43)* | Prospective Multicenter Cohort  Study | European Study “EPIC”: Denmark, France, Germany, Greece, Netherlands, Spain, Norway, Sweden, United Kingdom, Italy: Varese (Lombardia), Torino (Piemonte), Firenze (Toscana), Napoli (Campania), Ragusa (Sicilia) | Initial 47,021 (Varese=11,809, Torino=10,528, Firenze=13,436, Napoli=4,984 women only; Ragusa=6264).  Investigated: 44,099 | 1993-98 | 10,9 (mean) | 1.Free from MCV, 2. 35-74 years old | 54,5 | 31,60% | To investigate the relationship between glycemic index and glycemic load with the risk of stroke |
| Agnoli et al., 2011(25)* | Prospective Multicenter Cohort  Study | European Study “EPIC”: Denmark, France, Germany, Greece, Netherlands, Spain, Norway, Sweden, United Kingdom, Italy: Varese (Lombardia), Torino (Piemonte), Firenze (Toscana), Napoli (Campania), Ragusa (Sicilia) | Initial: 47,021 (Varese = 11,809; Turin = 10,528; Florence = 13,436; Naples = 4,984 women only; Ragusa = 6,264).  Investigated: 40,681 | 1993-98 | 7,9 (mean) | 1.Free from MCV, 2. 35-74 years old | 54,5 | 31,60% | To investigate the association between stroke and adherence to four a priori defined diet types |
| Pala et al., 2019 (39)* | Prospective Multicenter Cohort  Study | European Study “EPIC”: Denmark, France, Germany, Greece, Netherlands, Spain, Norway, Sweden, United Kingdom, Italy: Varese (Lombardia), Torino (Piemonte), Firenze (Toscana), Napoli (Campania), Ragusa (Sicilia) | Investigated:  45,009 | 1993-98 | 14,9 (mean) | 1.Free from MCV, 2. 35-74 years old | 54,5 | 31,10% | To investigate the association between the consumption of various foods and mortality |
| Keys et al., 1984 (31)** | Prospective Multicenter Cohort Study with Record Linkage | European Seven Countries Study: United States, Finland, Netherlands, Italy, Croatia (former Yugoslavia), Serbia (former Yugoslavia), Greece, Japan; Italy:  Crevalcore Emilia-Romagna), Montegiorgio (Marche), ferrovie di Stato di Roma (Lazio) | Investigated:  2,400 (Crevalcore=956, Montegiorgio=708, Roma=736) | 1959-60 | 15 | 1.Males  2.Free from MCV, 3.40-59 years old | 49,5 | 100% | To analyse 15-year mortality due to CHD and all causes |
| Menotti et al., 1990  (34)** | Prospective Multicenter Cohort Study with Record Linkage | European Seven Countries Study: United States, Finland, Netherlands, Italy, Croatia (former Yugoslavia), Serbia (former Yugoslavia), Greece, Japan; Italy: Crevalcore Emilia Romagna), Montegiorgio (Marche | Investigated:  1,712 (Crevalcore=993; Montegiorgio=719) | 1960 | 25 | 1.Males  2.Free from MCV, 3.40-59 years old | 49,5 | 100% | To analyse 25-year mortality due to CHD and all causes |
| Menotti et al.,  2002  (35)** | Prospective Multicenter Cohort Study with Record Linkage | European Seven Countries Study: United States, Finland, Netherlands, Italy, Croatia (former Yugoslavia), Serbia (former Yugoslavia), Greece, Japan; Crevalcore Emilia-Romagna), Montegiorgio (Marche), ferrovie di Stato di Roma (Lazio) | Investigated:  2.480 | 1957-1964 | 25 | 1.Males  2.Free from MCV, 3.40-59 years old | 49,5 | 100% | To investigate 25-year mortality trends for CHD and all causes. |
| Menotti et al., 1991  (38)** | Prospective Multicenter Cohort Study with Record Linkage | European Seven Countries Study: United States, Finland, Netherlands,  Croatia (former Yugoslavia), Serbia (former Yugoslavia), Greece, Japan; Italy:  Crevalcore Emilia-Romagna), Montegiorgio (Marche), ferrovie di Stato di Roma (Lazio) | Investigated:  2.088 | 1960-62 | 20 | 1.Males  2.Free from MCV, 3.40-59 years old | 49,5 | 100% | To investigate the role of changes in systolic blood pressure during the first 10 years of follow-up as a potential predictor of fatal events recorded in the subsequent 10 years of follow-up |
| Menotti et al, 2015  (36)** | Prospective Multicenter Cohort Study with Record Linkage | European Seven Countries Study: United States, Finland, Netherlands, Croatia (former Yugoslavia), Serbia (former Yugoslavia), Greece, Japan; Italy:  Crevalcore Emilia-Romagna), Montegiorgio (Marche | Investigated:  1,677 | 1960 | 50 | 1.Males  2.Free from MCV, 3.40-59 years old | 49,5 | 100% | To investigate the potential correlation between the lifestyle and the incidence of CVD |
| Puddu and Menotti, 2015  (41)** | Prospective Multicenter Cohort Study with Record Linkage | European Seven Countries Study: United States, Finland, Netherlands, Croatia (former Yugoslavia), Serbia (former Yugoslavia), Greece, Japan; Italy: Crevalcore Emilia Romagna), Montegiorgio (Marche | Investigated:  1,677 | 1960 | Over 50 | 1.Males  2.Free from MCV, 3.40-59 years old | 49,5 | 100% | To describe the natural history of the incidence of CHD. |
| Menotti and Menotti, 2019  (37)** | Prospective Multicenter Cohort Study with Record Linkage | European Seven Countries Study: United States, Finland, Netherlands, Croatia (former Yugoslavia), Serbia (former Yugoslavia), Greece, Japan; Italy:  Crevalcore (Emilia-Romagna), Montegiorgio (Marche | Investigated:  1,677 | 1960 | 50 | 1.Males  2.Free from MCV, 3.40-59 years old | 49,5 | 100% | To describe the natural history of the incidence of CVD. |
| D’Alessandro et al., 1992  (28) | Single-center observational population study (Aosta Hospital), with Record Linkage | Valle d'Aosta | Investigated:  114,325 | 1989 | 1 | The entire population permanently residing in the Aosta Valley with a first episode of stroke, TIA, or recurrent stroke (with WHO diagnosis). | All age groups | 49,50% | To determine the incidence rate, risk factors, and prognosis of stroke |
| Lauria et al., 1995 (32) | Prospective Multicenter Cohort Study with Record Linkage | Province of Belluno (Veneto) | Investigated:  211,389 | 1992-1993 | 1 | All residents in the province of Belluno. | All age groups | 47,70% | To determine the incidence, risk factors, and mortality of first stroke |
| Carolei et al., 1997  (26) | Prospective Multicenter Cohort Study with Record Linkage | Province of L’Aquila (Abruzzo) | Investigated:  297,838 | 1994 | 1 | All residents in the province of Aquila | All age groups | / | To determine the incidence of stroke and correlate it with population aging |
| Petrelli et al, 2006  (40) | Single-center retrospective population study with Record Linkage | Torino (Piemonte) | Initial: 529,284 | 1997 | 5 | 1.All residents in Turin at the beginning of 1997 2. aged between 35 and 74 years. | 35-74 | 47,90% | To investigate the potential role of contextual and individual socioeconomic determinants on coronary event mortality. |
| Manobianca et al., 2008  (33) | Prospective Multicenter Cohort Study with Record Linkage | Acquaviva delle Fonti, Casamassima (Puglia) | Investigated:  38,735 | 2001-2002 | 2 | All residents with a first episode of stroke in a defined rural geographic area: Acquaviva delle Fonti, Casamassima (Puglia). | All age groups | 49,20% | To determine the incidence and 28-day mortality of stroke |
| Corso et al., 2009  (27) | Single-center prospective population study with Record Linkage | Valle d'Aosta | Investigated:  123,748 | 2004-2005 | 2 | The entire population residing in the Aosta Valley with a first episode of stroke (with WHO diagnosis). | All age groups | 49,20% | To determine the incidence of stroke, mortality rate, and disability at 1 year |
| D'Ovidio et al., 2015  (30) | Single-center prospective population study with Record Linkage | Torino (Piemonte) | Investigated:  109,264 | 2002-2010 | 8 | 1.All women residing in Turin 2. aged 25-50 years,  3. free from coronary artery disease,  4.who were living alone or married, with or without children | 25-50 | 0% | To estimate whether the risk of coronary artery disease is correlated with work and childcare responsibilities (including differences based on the gender of the children) |
| De Bont et al., 2023  (29) | Prospective Multicenter Cohort Study with Record Linkage | European study with Germany, the Netherlands, Sweden, Catalonia, and Greece; Rome (Lazio) | Investigated:  1,539,784 | 2011 | 7 | Residents in Rome aged >37 years. | 59 | 44,7% | To evaluate the association between stroke incidence in Europe and long-term exposure to various environmental pollutants |
| * EPICOR : Italian cohort of the European Prospective Investigation into Cancer and Nutrition (EPIC)  ** Seven Country study  MCV: Major Cardiovascular Events; CVD: Cardiovascular Disease; CHD: Coronary Heart Disease; TIA: Transient Ischemic Attack; WHO: World Health Organization  **Single-Center:** data from one facility/registry in a defined area. **Multicenter:** data aggregated from multiple facilities/registries across sites. | | | | | | | | | |
